# Papillary Thyroid Micro Carcinoma: The Incidence of High-Risk Features and Its Prognostic Implications

**DOI:** 10.3389/fendo.2019.00074

**Published:** 2019-02-15

**Authors:** Rui Gao, Xi Jia, Yiqian Liang, Kun Fan, Xiaoxiao Wang, Yuanbo Wang, Lulu Yang, Aimin Yang, Guangjian Zhang

**Affiliations:** ^1^Department of Nuclear Medicine, The First Affiliated Hospital of Xian Jiaotong University, Xi'an, China; ^2^Department of Thoracic Surgery, The First Affiliated Hospital of Xian Jiaotong University, Xi'an, China

**Keywords:** papillary thyroid micro carcinoma, risk of recurrence, prognostic factors, total thyroidectomy, 131I therapy

## Abstract

**Background:** The current management of papillary thyroid micro carcinoma (PTMC) has become more conservative. However, high-risk characteristics that can only be revealed post-surgically exist. Patients and clinicians need to estimate the risks and understand the prognostic meaning of these factors.

**Methods:** We retrospectively analyzed 246 consecutive patients with PTMC who underwent surgery at our institution between 2015 and 2017. Clinical and histopathological parameters that may indicate recurrent disease were investigated. The responses to therapy in cases with different risks of recurrence were analyzed.

**Results:** A total of 79.26% (195/246) of patients received total thyroidectomy (TT), of whom 177 (90.77%) also received central lymph node dissection. Radioiodine ablation (RAI) was applied in 64.23% (158/246) of patients. Intermediate-high risk features were identified in 27.64% (68/246) after primary treatment. After a median follow-up of 18 months (range, 6–39 months), 121 of 158 (76.58%) patients who received TT+RAI were evaluated as an excellent response. An incomplete response (IR) was observed in 14.56% (23/158) of this group of PTMC. Multivariate analysis identified extra thyroid extension (*P* = 0.001) and intermediate-high risk stratification (*P* = 0.014) as significant and independent risk factors for an IR.

**Conclusions:** A total of 27.64% of PTMC cases evaluated as a low risk of recurrence pre-surgery showed intermediate to high risk disease post-surgery, and this leads to a higher rate of IR.

## Introduction

Papillary thyroid micro carcinoma (PTMC), which is defined as papillary thyroid carcinoma measuring 1 cm or less, have increased at the greatest rate in recent years worldwide (1–3). The concomitant increase in PTMC and Hashimoto's thyroiditis (HT) was suspected to predispose one another. A possible protective role of HT on the biological behavior of PTMC was indicated (4, 5). As PTMC without high-risk features showed an excellent prognosis, the traditional management approach of immediate thyroid surgery for PTMC is being reconsidered (6–9). The 2015 guidelines of the American Thyroid Association (ATA) now advise a risk-directed approach in the management of thyroid cancer (10). Lobectomy, or even an active surveillance protocol, which involves serial imaging studies and thyroglobulin (Tg) measurements, is now suggested for PTMC without known preoperative risk factors (8–12). Studies indicated a low rate of growth and metastatic potential in properly selected patients subjected to active surveillance (8, 9).

However, risk factors of PTMC that can be assessed preoperatively are relatively limited for clinicians to decide the extent of surgery dissections. A high prevalence of high-risk characteristics that can only be revealed at the final pathology is found in low-to-intermediate risk differentiated thyroid carcinoma (13, 14). Studies showed that nearly half of low-risk tumors may harbor pathological characteristics that are suggestive of a more radical treatment (i.e., total thyroidectomy, neck dissection, and subsequent radioiodine ablation) to lower the reoperation rate and achieve a better prognosis (15, 16). How frequently are the high-risk pathological features that may change the therapeutic strategy present in PTMC, and more importantly, how these factors may affect prognosis of the patients is unknown. Thus, our study was designed to retrospectively analyze the prevalence of these risk factors in a consecutive cohort of PTMC patients and understand the prognostic meaning of these factors.

## Materials and Methods

### Study Cohort

A retrospective analysis of patients who underwent surgical operations for PTMC at the First Affiliated Hospital of Xi'an Jiaotong University between January 2015 and June 2017 was performed. We excluded patients who had any of the following known high-risk characteristics preoperatively as per 2015 American Thyroid Association (ATA) guidelines (10): (i) clinically apparent lymph node metastases, (ii) distant metastases, and (iii) a history of radiation or a positive family history. Bilateral nodularity was not used as an exclusion criterion.

Study participants underwent total thyroidectomy (TT) or lobectomy, with/without prophylactic central neck or therapeutic central neck and lateral neck dissection as per the clinical situations (17). In patients who showed the presence of unfavorable factors in post-surgery pathological studies (i.e., extra thyroid extension, multifocality, lymph node metastasis, post-operative residual disease in the neck, and age older than 50 years), radioiodine ablation (RAI) was performed (10, 11). RAI was performed within 1 month after surgery in the hypothyroidism condition. The serum thyroid stimulating hormone (TSH) levels were >30 uIU/mL at RAI after levothyroxine withdrawal.

After primary treatment, all the patients received levothyroxine at TSH-suppressive doses and were periodically (every 3–6 months) followed up with measurement of thyroid hormones, TSH, serum thyroglobulin (Tg) and anti-thyroglobulin antibody (Tg-Ab). Additional diagnostic imaging tests such as 131I whole-body scan (WBS), neck ultrasonography and/or contrast enhanced computed tomography were performed every 6–12 months, as needed (10, 11, 18). Information on the last follow-up and the date of disease recurrence/metastasis was also collected. This study was carried out in accordance with the recommendations of the Ethics Committee of The First Affiliated Hospital of Xi'an Jiaotong University. All subjects gave written informed consent in accordance with the Declaration of Helsinki. The protocol was approved by the Ethics Committee of The First Affiliated Hospital of Xi'an Jiaotong University.

### ATA Risk Stratification

The preoperative risk stratification was mainly based on patient details, including age, sex, preoperative fine needle aspiration cytology (FNAC), and the preoperative ultrasound characteristics. Postoperative patients were re-classified into intermediate-high or low risk categories as per the 2015 ATA Risk Stratification (10). Briefly, intermediate-high risk PTMC was defined as extra thyroidal extension, RAI avid metastatic disease in the neck, >5 positive lymph nodes (at least 0.2 cm in largest dimension), >3 vascular invasion, aggressive histological variants, and/or synchronous distant metastases (iodine avid or non-avid). The remaining patients were classified as low risk.

### Response to Therapy

Based on follow-up data, patients were classified as follows. An excellent response was defined as negative imaging and either serum TSH-suppressed thyroglobin (sup-Tg) levels <0.2 ng/mL or stimulated Tg (sti-Tg) levels <1 ng/mL. A biochemical/structural incomplete response (IR) was defined as negative imaging with sup-Tg levels ≥0.2 ng/mL, sti-Tg levels ≥10 ng/mL, rising Tg-Ab levels, or structural/functional evidence of disease with any Tg level. An indeterminate response (InR) was defined as a serum sti-Tg ≥ 1 and <10 ng/mL, and/or the presence of non-specific imaging abnormalities (10).

### Statistical Analysis

Continuous variables are shown as median (range). Categorical data are shown as frequencies and percentages. χ^2^ test was used for comparison of categorical variables. Risk factors associated with migration of risk stratification were analyzed with univariate analysis. As differences existed between pre-surgery imaging tests and post-surgery pathology studies, the pre-surgery ultrasonographic and post-surgery pathologic findings were both included in the analysis. Factors to predict persistent disease were analyzed by univariate and multivariate logistic regression and included the following characteristics: age, sex, tissue background, tumor size, capsular invasion, vascular invasion, extra thyroidal extension, multifocality, central compartment lymph node metastasis (CLNM), 131I WBS avidity, and risk of recurrence. Variables associated with persistent disease with a *P-*value of <0.10 in the univariate analysis were included in the multivariate regression analysis. A *P* < 0.05 was considered statistically significant. SPSS 13.0 software (SPSS Inc., Chicago, IL, USA) was used for statistical analysis.

## Results

### Patients' Characteristics

A total of 1,160 patients with differentiated thyroid carcinoma (DTC) were reviewed and 246 PTMC cases were found. The clinical and histological characteristics of the patient population are shown in Table 1. The overall median age was 46 years and women represented the 73.17% of the study population.

**Table 1:** Initial characteristics and primary treatment of 246 PTMC patients.

		***n* (%)**	**Median (range)**
Age, y			46 (14–71)
Sex	Male	66 (26.83%)	
	Female	180 (73.17%)	
Histology	cPTC	240 (97.56%)	
	FvPTC	5 (2.03%)	
	Hurthle cell	1 (0.4%)	
Maximum of diameter, mm			0.8 (0.1–1)
Multifocality	Absent	151 (63.71%)	
	Present	86 (36.29%)	
LN metastasis	Absent	108 (43.90%)	
	Present	109 (44.31%)	
	CND only	95 (87.16%)	
	CND+LND	14 (12.84%)	
	Not defined	29 (11.79%)	
Capsular infiltration	Absent	75 (32.33%)	
	Present	157 (67.67%)	
	Not defined	14 (5.69%)	
Extra thyroidal extension^*^	Absent	225 (91.46%)	
	Present	21 (8.54%)	
Vascular invasion	Absent	103 (41.87%)	
	Present	27 (10.98%)	
	Not defined	116 (47.15%)	
TNM stage	I	226 (91.87%)	
	II	20 (8.13%)	
Surgery	Lobectomy	51 (20.73%)	
	CND only	37 (72.55%)	
	CND+LND	3 (7.5%)	
	TT	195 (79.27%)	
	CND only	157 (80.51%)	
	CND+LND	20 (10.26%)	
RAI	No	88 (35.77%)	
	Yes	158 (64.22%)	
	Dose (GBq)		4.44 (2.96–20.35)
RAI avid metastasis		48 (48/158, 30.38%)	
	LN metastasis	42 (87.5%)	
	Lung metastasis	5 (10.42%)	
	Bone metastasis	1 (2.08%)	
Tissue background	Simple goiter	10 (4.07%)	
	Multinodular goiters	72 (29.27%)	
	Thyroiditis	43 (17.48)	
	Grave's disease	7 (2.85%)	
	Not defined	114 (46.34%)	

*PTMC, papillary thyroid micro carcinoma; cPTC, classic papillary carcinoma; fvPTC, follicular variant papillary thyroid cancer; TT, total thyroidectomy; LN, lymph node; CND, central compartment dissection; LND, lateral neck dissection (level II-IV); RAI, radioiodine ablation*.

* *Present of infiltration of the tumor to the perithyroid soft tissues, or sternothyroid muscle, or surrounding organs, such as the trachea, esophagus, recurrent laryngeal nerve, jugular vein, or vagus nerve*.

Primary management of PTMC consisted of surgery in all patients, followed by RAI in cases showing unfavorable factors in post-surgery studies (Table 1). TT was performed in 195 patients (79.27% of all cases). Central neck dissection (CND) was carried out in 157 (80.51%) of them and CND + lateral neck dissection (LND, level II-IV) was performed in 20 (10.26%). Lobectomy was performed in 51 patients (20.73% of all cases). Of them, 37 (72.55%) also received CND and 3 (7.5%) received CND + LND of the lesion side. In 158 patients (81.03% of TT cases), RAI was also administered. The administered radioiodine activity ranged from 2.96 to 20.35 GBq (median 4.44 GBq).

**Figure 1:**
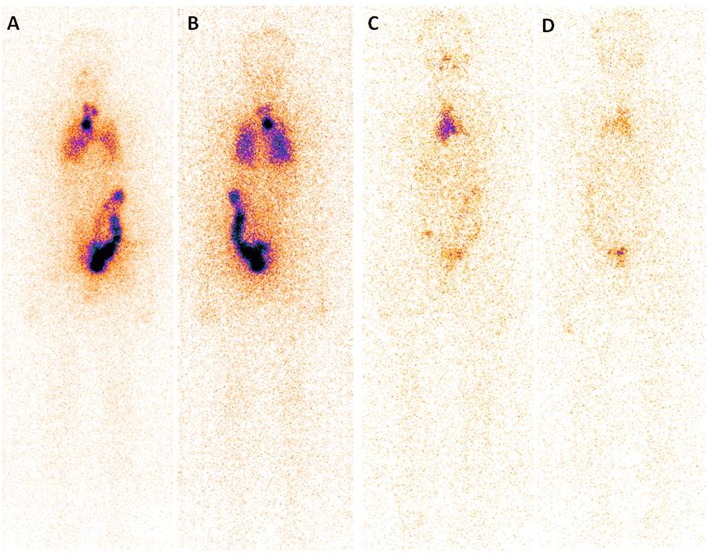
Post-treatment 131I scan (Rx-WBS) after first dose of 131I (3.7 GBq) demonstrates abnormal 131I activity in neck and thoracic regions **(A,B)**. The case with raised serum thyroglobulin level after total thyroidectomy + central neck dissection (TSH stimulated Tg 7.99 ng/mL) had received a total cumulative dose of 17.02 GBq of 131I at time of the forth Rx-WBS imaging **(C,D)**. Though no abnormal tracer activity is demonstrated in neck and pulmonary area, persistent tracer activity is seen in mediastinal region.

A post-surgery pathological study showed that classic PTMC was the most common histological subtype (97.56% of all cases) and the median tumor size was 8.0 mm (range, 0.1–10 mm). Notably, in 86 patients (36.29% of all cases), the tumor was multifocal, and it was not encapsulated in 232 (67.67% of all cases), with extra thyroidal extension found in 21 (8.54% of all cases). Lymph node metastases were found in 44.31% of all cases (109/246), with 35.61% (517/1452 from CND) and 23.53% (48/204 from LND) of the dissected lymph nodes was found to harbor metastatic disease (Table 1). Finally, at WBS, lung metastases were found in 5 patient and bone metastases were found in one patient (Figure 1).

In 158 patients that received RAI, the median post-operative TSH level was 75.7 uIU/mL. In this condition, the Tg level was elevated in the absence of Tg-Ab in 72 cases (median, 4.89 ng/mL; range, 1.78 to 211.6 ng/mL) and was 1 ng/mL in the absence of Tg-Ab in 19 cases. Detectable Tg-Ab was present in 22 cases (7 of which had elevated Tg values). Post-operative Tg values were not available in 45 patients.

### Distribution in ATA Risk Categories

In accordance with the current ATA recommendations (10), all the included PTMC cases were classified as a low risk of recurrence before surgery. Among them, 68 (27.64% of the cases) were classified into the intermediate-high risk category based on post-surgery findings (Table 2). 29.41% (20/68) was reclassified due to >5 positive lymph nodes and/or extra thyroidal extension, 51.47% (35/68) were due solely to RAI-avid metastasis, and 19.11% (13/68) were due to both factors.

**Table 2:** Response to primary treatment as per response to treatment category.

		**Lobectomy** ***n*** **=** **51**		**Total thyroidectomy** ***n=*** **195**			
**RAI**		**0**		**Without RAI** ***n*** **=** **37 (18.97%)**		**With RAI** ***n*** **=** **158 (81.03%)**	
**Risk of recurrence**^**a**^		**Low**	**Inter-high**	**Low**	**Inter-high**	**Low**	**Inter-high**
		47 (92.16%)	4 (7.84%)	35 (94.59%)	2 (5.40%)	96 (60.76%)	62 (39.24%)
Response to therapy^b^	ER	46 (97.87%)	4 (100%)	33 (94.29%)	1 (50%)	79 (82.29%)	42 (67.74%)
	bIR	/	/	/	/	2 (2.08%)	3 (4.84%)
	sIR	1 (2.13%)	0	2 (5.71%)	1 (50%)	4 (4.17%)	14^*^ (22.58%)
	InR	/				11 (11.46%)	3 (4.84%)

*CND, central compartment dissection; RAI, radioiodine ablation; ER, excellent response; bIR, biochemical incomplete response; sIR, structural incomplete response; InR, indeterminate response*.

a *Risk of recurrence evaluated after primary treatment*.

b *Response evaluated during follow-up*.

* *Compared with low-risk group who received TT+RAI*.

We further evaluated whether any preoperative features can help predict migration of risk stratification. Unfortunately, none of the pre-surgery factors were related with the migration of risk stratification in patients with PTMC (age, *P* = 0.896; sex, *P* = 0.105; ultrasonographic—tumor size, *P* = 0.291;—capsular invasion, *P* = 0.781; -multifocality, *P* = 0.692; and—lymph node metastasis, *P* = 0.575). As expected, multifocality, capsular invasion, extra thyroidal extension, CLNM and 131I WBS avidity was closely related with risk stratification migration (*P* = 0.006, 0.006, <0.001, <0.001, and <0.001, respectively). Interestingly, we found that male gender (*P* = 0.006, odds ratio [OR] 2.894) and ultrasonographic tumor diameter (*P* = 0.024, OR 2.382) are risk factors for pathological CLNM.

### Response to Therapy

The patients were followed up for a median of 18 months (6–39 months). In patients whose surgery consists of lobectomy or those who have TT without RAI, a normal thyroid remnant may lead to detectable Tg levels, which may result in a false diagnosis of biochemical IR (10). Therefore, response to therapy was only evaluated in the 158 patients who received TT + RAI to determine how high risk factors may affect the prognosis of PTMC. An excellent response was achieved in 121 (76.58%) patients, i.e., these patients had no clinical, biochemical, or structural evidence of disease identified in follow-up studies. A biochemical/structural IR were found in 14.56% (23/158) of these patients. Fourteen of the cases demonstrated InR due to slightly elevated sti-Tg levels (8.86%, Table 2). Notably, four patients had structural evidence of recurrence among the 88 cases who did not receive RAI (4.55%, Table 2).

Of the 23 patients who showed an IR after TT + RAI, 5 were due to consistently elevated Tg levels after treatment (biochemical IR) and the other 18 were due to recurrent/metastatic disease as shown in imaging tests, including WBS, neck ultrasonography and contrast-enhanced CT during the follow-up (structural IR). We found a significantly higher incidence of an IR in patients who showed an intermediate-high risk of recurrence compared with those who showed a low risk (*P* = 0.002, Table 2).

Four out of the six IR cases with low risk were structural IR. Two of them underwent lateral compartment neck dissection and another course of RAI (dose, 4.8 and 5.5 GBq, respectively). The remaining 2 with elevated sti-Tg levels (38.4 and 36.9 ng/mL, respectively) were only closely followed up (Tg evaluation and ultrasound every 3 months). Their sup-Tg kept stable during the follow-up. Most of the IR cases at intermediate -high risk were structural IR (14/17). Six of them received second surgery followed by RAI and the other 8 received another course of RAI only. Except the 6 cases with distant metastasis, the other 8 patients were alive with no evidence of structural disease by the end of the study. The sti-Tg level of the biochemical IR cases with intermediate-to-high risk PTMC was 13.5, 31.6, and 59.6 ng/mL, respectively. They were administered a second course of RAI and the sti-Tg level decreased below 10 ng/mL in 2 of them. The remaining one showed a negative sup-Tg during the follow-up. As for the cases with InR due to elevated sti-Tg levels (median 2.40, range 1.09–7.3 ng/mL, *n* = 14), they were applied no further treatment but close follow-up (Tg evaluation and ultrasound every 3 months). Their sup-Tg was all negative during the follow-up.

We compared the clinicopathologic features of PTMC between IR and excellent response cases. PTMC with an IR was significantly more frequently found in patients with extra thyroidal extension and intermediate-high risk stratification (*P* = 0.001, = 0.014, respectively, Table 3).

**Table 3:** Clinicopathologic features of PTMC associated with incomplete response.

**Variable**		**IR/All TT+RAI patients** **23/158 (%)**	**Univariate analysis** **OR (95% CI)**	***P*-value**	**Multivariate analysis** **OR (95% CI)**	***P*-value**
Age	<55	15/127 (11.81)	1	0.626		
	≥55	8/31 (25.81)	0.655 (0.479–3.396)			
Sex	Female	15/112 (13.39)	1	0.868		
	Male	8/46 (17.39)	0.921 (0.349–2.431)			
Tg before operation (ng/mL)	≤10	7/80 (8.75)	1			
	>10	16/78 (20.51)	1.535 (0.585–4.028)	0.384		
Tissue background	ND	12/72 (16.67)	1	0.442		
	Grave's disease	2/3 (66.67)	1.354 (0.626–2.930)			
	Multinodular goiters	5/52 (9.62)	0.794 (0.463–1.361)	0.401		
	Thyroiditis	5/31 (16.13)	0.839 (0.502–1.403)	0.503		
Tumor diameter	≤5 mm	6/37 (16.22)	1	0.161		
	>5 mm	17/121 (14.05)	1.429 (0.306–6.663)			
Capsular infiltration	Absent	8/43 (18.60)	1	0.354		
	Present	15/110 (13.64)	1.459 (0.656–3.246)			
	ND	0/5 (0)	/	/		
Extra thyroidal extension	Absent	17/143 (11.89)	1	0.002	1	0.015
	Present	6/15(40)	4.682 (1.599–13.705)		5.462 (1.398–21.337)	
Vascular invasion	Absent	9/62 (14.52)	1	0.907		
	Present	3/19(15.79)	1.172 (0.541–2.482)			
	ND	11/77(14.29)	0.876 (0.298–3.661)	0.974		
Multifocality	Absent	14/85 (16.47)	1	0.030	1	0.065
	Present	9/73 (12.33)	2.566 (1.095–6.012)		2.829 (0.937–8.543)	
CLNM	≤5	16/125 (12.8)	1	0.016	1	0.337
	>5	4/21 (19.05)	3.561 (1.263–10.043)		1.909 (0.510–7.142)	
	ND	3/12 (25)	0.784 (0.266–4.732)	0.50	/	/
RAI avid metastasis	No	9/108 (8.33)	1	<0.001	1	0.081
	Yes	14/50 (28)	8.320 (2.796–24.760)		3.887 (0.843–17.929)	
Risk of recurrence	Low	7/118 (5.93)	1	<0.001	1	0.016
	Intermediate-high	16/40 (40)	10.783 (2.990–38.887)		8.159 (1.474–45.160)	

*CLNM, central compartment lymph node metastasis; ND, not defined; RAI, radioiodine ablation; Tg, thyroglobin*.

## Discussion

There is considerable debate regarding the optimal therapeutic approach of PTMC. Active surveillance (AS) management approach was suggested as an option in properly selected low risk patients, who showed no pre-operative known unfavorable features. For AS to be successful, a critical assessment of the tumor characteristics is required (8–12). However, discriminating patients with aggressive factors from those without these factors before surgery is barely possible, as some high-risk characteristics are only apparent upon pathology examination or post-operative radioiodine WBS (13–16). In this retrospective study, some high-risk factors for patients with PTMC, such as tumor multifocality, microscopic extra thyroidal extensions, incidental lymph node metastases, and distant metastasis were unknown until post-surgery assessment. Consequently, 27.64% of the cases migrated from the low-risk category to the intermediate-high risk category. This is in line with recent reported data of 1–4 cm DTC, which showed that approximately 40–50% of low risk patients require more radical treatment based on pathological characteristics post-surgery (13–16). In fact, this is the problematic reasoning of the 2015 ATA guidelines, which advise that decisions about the operative extent of the thyroidectomy should be based on risk factors available only after the operation (10). Better preoperative risk stratification is required based on these findings.

There was a high percentage of capsule invasion (67.67%) and lymph node metastases (44.31%) in our PTMC population (13, 14, 19, 20). The high prevalence of lymph node involvement might be related to the high proportion of patients who received neck dissections (88.21%, 217/246). Though debated in the literature over the past decades, prophylactic CND is widely performed in PTMC to allow more accurate TNM staging and risk stratification, and thus decide the post-operative management (21–24). The incidence of CLNM is reported to be highly prevalent in cN0 PTMC and implementation of prophylactic CND was supposed to lower the incidence of residual lymph node metastasis and improve the overall prognosis (23, 24). As male sex and ultrasonographic tumor diameter are shown to be independent risk factors of CLNM in these PTMC patients, surgeons would be suggested to consider these risk factors when deciding the extent of surgical dissections (25, 26).

81.09% (158/195) of the PTMC patients who received TT had RAI owing to the presence of unfavorable factors (15). Interestingly, having more than five positive lymph nodes and/or gross extra thyroidal extension was infrequently the only cause for intermediate-high risk classification. Most (59.37%, 38/62) cases were categorized as intermediate-high risk because of metastasis detected by a post RAI WBS, which included 6 cases of distant metastasis(9.68%, 6/62). The effectiveness of 131I ablation in patients with PTMC is controversial (27–29). Though currently no study precisely indicate which patients with PTMC may benefit from post-operative RAI, examination results obtained pre- or post RAI provides information for dynamic risk stratification, and thus helps determine the long-term follow-up strategy (30). In line with our data, a prospective study revealed that diagnostic 131I scans identified unsuspected nodal metastases in about 30% of the DTC patients initially assigned pathologic N0 or Nx (30). Thereafter, a risk stratification predicated solely on surgical pathology information might miss −30% of the metastatic disease, which support a role of 131I WBS in dynamic PTMC risk stratification systems (30–34).

Of the 158 patients who received TT+RAI, 39.24% (62/158) transferred from low risk to intermediate-high risk stratification. Eight out of the 62 patients (12.90%) were diagnosed as loco regional recurrence or metastasis, which was identified as the most prevalent factor for high risk stratification. After treatment with high dose RAI (3.7 GBq), patients with intermediate risk DTC were supposed to have a low loco regional recurrence rate. However, about 20% of these patients was reported to have persistent/recurrent diseases (35). This indicated that a certain percentage of PTMC should be considered at high risk, characterized by aggressive behavior like that of classical DTC (17, 36). Identification of these patients is important because they may require a radical therapeutic approach. Since they may not respond well to treatment, close follow-up with structural or functional imaging was also suggested (37, 38).

Among patients who showed an IR, 21.74% (5/23) of cases were due to elevated Tg levels. Further treatment was suggested in DTC cases with increased serum Tg levels after TT and successful RAI, especially when sti-Tg levels are >10 ng/mL and/or sup-Tg levels are >2 ng/mL, because of possible existent recurrent and/or metastatic disease (39, 40). It is of note that two low risk PTMC cases with sti-Tg levels higher than 35 ng/mL but no evidence of structural recurrence/metastasis received no further treatment; their Tg levels were stable during the follow-up. Thus, we would suggest that biochemical IR patients in low risk PTMC might have their risk of having recurrent disease decreased, as predicted by consecutive Tg evaluations (41–43). It is also true for the cases of indeterminate response. The 14 cases with slightly elevated Tg levels was applied no further treatment but follow-up. As they showed negative image and stable Tg levels during follow-up, their risk of having recurrent disease also decreased.

In previous studies, the most relevant clinical and histological risk factors for disease recurrence of PTMC were the presence of lymph node metastases at initial diagnosis, tumor multifocality, and the extent of primary thyroid surgery (44, 45). There is no agreement in the literature about the statistical significance of sex, age, and tumor size as prognostic factors for PTMC recurrence (44–46). Our analysis focusing on PTMC showed that only extra thyroidal extension and risk of recurrence were found to be independent risk factors for incomplete response after TT+RAI in PTMC. These results further supported a close follow-up in cases evaluated as intermediate-high risk after primary treatments.

There are some limitations in our study. The limitations include potential selection bias that may be present in any retrospective, single institutional series, and a lack of uniformity between surgeons/sonographers in reporting and surgical procedures. Furthermore, patients were only followed up for a limited time, which may not reflect long-term survival of the disease. Additionally, the response to therapy was only evaluated in patients who received TT + RAI, while only patients who showed high risk factors post-surgery had RAI. Therefore, the rate of an IR in this study may have been overestimated. Finally, *BRAF*^*V600E*^status was not considered because mutational analysis is not routinely performed in our institution. Including this variable may increase the possibility of migration of the risk of recurrence.

In summary, more than one fourth of PTMC cases evaluated as having a low risk of recurrence pre-surgically may show intermediate-high risk disease post-surgically. A total of 15% of the patients who received TT+RAI in our study showed an incomplete response to therapy and most of them were intermediate risk disease. Radical therapeutic approach and close follow-up may be suggested in these PTMC patients.

## Data Availability

The raw data supporting the conclusions of this manuscript will be made available by the authors, without undue reservation, to any qualified researcher.

## Author Contributions

RG and GZ contributed to the conception and design of the work. XJ and YL participated to data analysis and text editing. KF, XW, YW, and LY participated to data collection and patients' follow-up. AY contributed to text revision.

### Conflict of Interest Statement

The authors declare that the research was conducted in the absence of any commercial or financial relationships that could be construed as a potential conflict of interest.
